# Embodying two shores of the Mediterranean Sea: the liminal masculinity of minors migrating alone to Spain

**DOI:** 10.3389/fsoc.2024.1420112

**Published:** 2024-08-08

**Authors:** Bakea Alonso, Valentina Longo, Álvaro Ruiz Garriga

**Affiliations:** ^1^Cepaim Foundation, Madrid, Spain; ^2^X-MEN Project Researcher and Trainer, Sevilla, Spain

**Keywords:** migrant minors, violence, masculinities, liminal spaces, social intervention, frontiers

## Abstract

The article aims to provide an analysis based on the practical experience of social intervention in violence prevention with migrant minors arriving alone in Spain. In order to offer keys and recommendations, and based on situated knowledge, we provide insights on how to approach the intervention framework from a methodological point of view using liminal spaces as border and transition places that need to be named and taken into consideration for a transformative work. Different metaphorical borders, characterized by tension and potential change, are analyzed from a gender and intersectional perspective. We discuss the Law-border and the tension between protection they receive as minors and exclusion/politics of suspicion they receive as migrants. The Age-border (children/men) is discussed taking into account the different gender regimes they cross. The Color-border: here issues of racism and colonialism are considered. Finally, we discuss the Masculinity-border and the tension between reception and the reproduction of violence. We use the concept of liminal masculinities as a specific state or form that subordinate or marginalized masculinity adopts in migrant minors, suspended legally, functionally, and biographically, among others borders.

## 1 Introduction and context

In a conventional sense, borders refer to geographical and political boundaries between countries or regions. However, the concept has become an increasingly plastic one, used in common parlance and across various disciplines, such as literature, sociology, journalism, etc. From an intersectional perspective, these borders can be places where people experience different levels of discrimination and violence, even death, because of their skin color, gender, sexual orientation or socio-economic status, as well as due to other systems that create inequality (Vaughan-Williams, 2009). More specifically, we will speak about border spaces as liminal spaces. Etymologically, liminality derives from the Latin *limen—minis*, which literally means threshold. The term liminal space is used to describe an “in-between condition,” a transitional space that aptly describes the lives of the young people we are talking about. Van Gennep (1960) studies liminal space in relation to the changes a person experiences within his or her own social system, which are witnessed and celebrated through rites of passage.

These spaces are inherently ambiguous and characterized by uncertainty and, at the same time, the possibility of transformation. In this article, we will use the plasticity of liminal spaces as a guiding principle in order to explore different aspects of the embodied experiences of migrant minors when crossing the Strait of Gibraltar and migrating alone to Spain. In other words, we are going to investigate the experiences of migrant children and then lay bare the liminal spaces that operate both systemically and subjectively based on the lessons learned in the X-MEN project.^1^

We the authors also find ourselves in a liminal zone and we apply the concept to ourselves in order to determine from where and how far we have traveled to arrive at this article, making explicit the practices, relationships and meanings we have experienced along the way. We draw on situated knowledge (Haraway, 1991) so as to avoid falling victim to absolute truths, but we do highlight the partial truths that we have gradually become aware of through research and workshops with young people carried out as part of the X-MEN project.

Sayad (2010) begins by considering migration as a total social fact^2^ that needs to be investigated in all its aspects, restoring unity and complexity to a fragmented subject and going beyond purely economic or demographic aspects. In our case, the social and media construction of young male adults migrating alone often focuses on the “social dangerousness” of these boys, whereas our framework is based on their vulnerability and, at the same time, their agency. Unaccompanied migrant children^3^ often find themselves in constrained spaces with different levels of porosity and permeability. The border space can be that of identities constructed in diaspora and through hybridity, accommodating multiple belongings and partial identity configurations. At the same time, these borders establish a line in order to protect consolidated political, social and symbolic spaces, constructing hierarchies in terms of (citizenship) rights and radical otherness in terms of the process of subjectification. When we get closer to it, the border not only functions as a dichotomous mechanism (black/white, woman/man, migrant/native, minor/adult), but also as a space which gives rise to violence, conflict and solidarity, crosscutting differences related to origin, skin color, class, gender, etc. (Razac, 2015).

Geopolitical borders can be seen a space of tension between the hardening of control devices and the challenge posed by migrants to such dispositifs allows us to think of borders as a disputed field, rather than simply a pacified mechanism of exclusion, since contemporary global processes are linked to a multiplication and profound transformation of borders. This ambiguity serves not to block migrants, but to filter and channel them. In this sense, border regimes aim to control who enters a country and direct them toward a labor market characterized by the proliferation of “global work”—that is, the precarious expansion and diversification of work in a global context (Mezzadra and Neilson, 2013).

We will speak of the *liminal masculinities* of migrant minors insofar as, although they already occupied a marginal position in the masculine hierarchy on a global scale (where being labeled as “black” man is not the same as being labeled as “Arab” man), when they enter the border dispositif they are trapped on the threshold, that is, in the tension between the new adultocratic and Eurocentric codes of infantilization and foreignization. Accordingly, we could argue that the liminal masculinities of migrant minors are a by-product or a perverse effect of the neo-coloniality of the late 20th and early 21st centuries (Empez, 2014).

Throughout the implementation of the X-MEN project, the analysis of gender is central, but always from an intersectional perspective. A person’s gender defines, in a significant way, their migratory experience and one of the borders considered in our analysis is liminal masculinity in relation to young men’s experiences of violence. This determines who migrates, the networks they use to be able to do so, the opportunities for integration and work at the destination, and the relationships with their country of origin. The expectations, relationships and power dynamics associated with being read as man, woman, or boy/girl affect all aspects of the migration process. Patriarchy, as the institutionalization of male dominance over women (Lerner, 1987), but also of hierarchy among men (Connell, 1995), is present in all human societies; however, in each culture it has its own manifestations and characteristics, but under the colonial influence of European patriarchy, which imposed its ethnocentric schemes of kinship, family and, therefore, gender (Casares, 2006; Grosfoguel, 2020; Beiras, 2023; Oyěwùmí, 2023) and which maintains its influence today through developmentalist policies and Islamophobic and neo-colonial interference (Adlbi Sibai, 2016; Grosfoguel, 2020).

Applying this to the situation of the young people with whom we carried out the workshops, it is clear that the male models they have been raised on, the place of women in their societies of origin and, in short, the *gender regime*^4^ (Connell, 1987) in which they have been raised differs, to a large extent, from the one they encounter when they arrive in Spain. However, it is essential to understand that neither the societies of origin nor the host societies are homogenous. Spanish society is not monolithic: on the one hand, there has been significant development in terms of equality policies and legislation that places it at the forefront of Europe in this regard but, on the other hand, there are still sectors of society which are reactive to the advancement of women’s rights, and which hold firm neo-sexist positions (Lorente, 2023). Nor is Moroccan society monolithic: according to a study conducted by Promundo and UN Women in 2017, more than three-quarters of Moroccan men support equal education for boys and girls, and more than half believe in equal rights to work. Moreover, more than 80% of both men and women reported being in favor of paid parental leave for fathers. However, it is also true that more than 60% of men (and almost half of women) still believe that wives should tolerate violence in order to keep the family together. Moroccan society itself is therefore also at a *border*, undergoing a transition in terms of culture, politics, values and social norms.

### 1.1 Minors migrating alone to Spain

The migratory phenomenon involving minors entering Spain dates back to the 1990s (Lázaro González, 2007) and has been growing in recent years. According to data collected in the Register of Unaccompanied Foreign Minors (Spanish State Prosecutor’s Office, 2023), at the end of December 2022, a total of 11,417 boys and girls (83% boys) were registered, showing an increase on 2021, when 9,294 minors were registered under the guardianship or foster care of the protection services. In terms of origin, the greatest number of these minors come from Morocco (1,235), followed by Algeria (402), Republic of Guinea (216), and Senegal (126).

From a terminological point of view, in this article, we will not use the acronym MENA (Spanish acronym for “unaccompanied foreign minor”), a technical and legal term, which in itself signaled a process involving the institutionalization and homogenization of the lives of young migrants and which has since become pejorative. This derogatory language is yet another facet of the process of the dehumanization and criminalization of migrant children, capitalized upon by forces from the extreme right, which thus contributes to the politics of suspicion and rejection of these children/young people. In her address to the Spanish Congress of Deputies on 21 November 2021,^5^ Violeta Assiego, then Director General for the Rights of Children and Adolescents, declared that her department would no longer use the term MENA, “since, unfortunately, today this acronym is used in a way which appears to refer more closely to a criminal gang and no longer fulfills its original objective, which was for particular terminological use in technical and legal documents,” thus removing the spotlight from the protection of migrant children.

Social organizations who deal with the rights of migrant children use several terms which reflect the concern that exists in relation to respect for these children and young people, emphasizing their age and their status as unprotected persons: unaccompanied migrant children; children who migrate alone; migrant adolescents without family members in Spain; children and adolescents in a situation of human mobility. As Viveros (2016) points out, it is a matter of deconstructing the normalizing and homogenizing categories that form part of the institutionalization process and restoring the complexity, multiplicity and singularities that migrant boys and girls embody.

As for the reception system provided by the Spanish authorities, these vary depending on the region (or *comunidad autónoma—*autonomous community). There are, on the one hand, centers specifically designed for these minors, others where they are placed with Spanish minors and, to a lesser extent, foster care in families. Also noteworthy are what are called Emergency or Urgency Centers, which are temporary residential facilities whose purpose is to provide reception and shelter services. These centers attend to the basic needs of these minors in terms of accommodation, food, health and clothing, as well as facilitating the assessment of their situation and safeguarding their rights. Many of these centers are run by NGOs, foundations or organizations linked to the church.

Moving on from this description of the context, we will then set out the methodological approach and the techniques used both in the research phase and in the implementation of the workshops with young male adults, the aim of which was to encourage them to reflect critically on the construction of masculinity, to become aware of the inequalities that affect women and themselves, and to avoid contributing to the reproduction of these inequalities. Subsequently, using the idea of the border as a guiding principle, we will present some reflections and discussion.

## 2 Notes from the north of Morocco and the south of Spain: materials and methods

In our research and intervention work, we have adopted an intersectional and intercultural approach that aims to be transformative because we understand that gender inequality can neither be isolated nor independent from other systems of oppression (Cubillos Almendra, 2015). As white feminist and anti-racist researchers and educators from the global North with higher education, we know that we have embedded Eurocentric and colonial views which we try to subvert with varying results. We also underline the partiality of our research and intervention: it is a quasi-exploratory experience, with blind spots and ample room for improvement. Finally, we recognize that we operate within a field -that of research applied to social intervention- which may aspire to dismantle narratives and practices of inequality and social injustice, but which, nevertheless, is embedded in structures (state, political, economic, etc.) which perpetuate this system, and which restrict intervention within narrow confines.

Viveros (2016) highlights the close relationship between theory, methodology and political outlook in the intersectional approach. This tension can constitute a starting point for social transformation or a perspective through which to build collective knowledge and practices, thus underlining the importance of the process. Thus, the initial design of the research and intervention has been modified over time according to the results of the ongoing fieldwork and as we detected the need to broaden and/or modify our approach.

### 2.1 Research phase

Since the aim of the project implied a socio-educative intervention with vulnerable African adolescents or young men in order to prevent the reproduction of gender-based violence (which we took in an intersectional point of view), our research had to be limited within the specific contextual and temporary frames of the intervention. Taking the complexities of the connections between different cultural constructions of masculinity, age, and migration as our starting point, we built an approach based on Grounded Theory. This involved collecting and interpreting information from selected informants during the process, as well as processing information from the entire intervention experience.

In the course of this research, we conducted one focus group and interviews with a range of stakeholders, including sector professionals, policymakers, experts, young migrants over 18, and vulnerable Moroccan youth. The fieldwork focused on the cities of Tetouan (Morocco) and Seville (Spain). We also incorporated learnings from an intervention program implemented in Seville. We interviewed an anthropologist and an educator who were engaged in both professional intervention/research and social activism. Their work aimed to support vulnerable young migrants beyond the constraints of institutional frameworks. Due to the difficulty in accessing underage migrants, we interviewed three young men from North Africa and West Africa who had previously been under guardianship and were participating in an emancipation program.

In addition, we organized a field trip (Eden et al., 2019) to Tetouan (Morocco) to attend the fourth international seminar on development cooperation in Northern Morocco entitled “Adolescent migrants without family connections: beyond the age of majority.”^6^ This meeting, organized by an independent organization of scholars and professionals that operate in both Spain and Morocco, was inspired by the goal of promoting the right to safe migration for minors. The meeting allowed us to connect with and learn from other Spanish and Moroccan organizations and professionals who work daily to address this issue in both countries. Prior to arriving in Tetouan, the organizers informed us about the possibility of conducting interviews or a group interview with some of the young people they work with. The host organization agreed to facilitate our participation in the research. During one of the meeting activities, we proposed that participants voluntarily take part in a focus group discussion. We explained the main aim of the research: “to understand what it means to be a man in Morocco and the implications for unaccompanied minors.” The participants found this topic interesting, relevant, and important. Initially, we had planned to conduct a focus group with only young men. However, this was a mixed-gender group of close friends who would not have understood the exclusion of the young women who were also eager to participate. Rather than impose our pre-determined methodological approach, we adapted to include the young women as well. Their participation ultimately provided a critical gendered perspective on the Moroccan masculine role, which was fundamental to the research.

Eventually, the group was composed by 10 young boys and girls from 17 to 23 years old. We also interviewed a Moroccan social agent, pioneer in the promotion of attention for homeless children at the community level, and a Spanish educator working with vulnerable children and youngsters in Tetouan and who had worked in hosting centers for young migrants in Andalusia (Table 1).

**Table 1:** Informers list.

Tetouan (North Morocco)	
Focus Group	10 Moroccan youngsters aged 17–23 years old, educated and supported by social organizations as individuals without family.
Interview L.T.	Moroccan community agent caring for orphans and homeless children.
Interview I.C.	Spanish social worker working with vulnerable children and teenagers in Tetouan and who had worked in hosting centers in Andalusia.
Sevilla (Andalusia, South Spain)	
Interview	Three young adults over 18, who arrived as unaccompanied minors, currently involved in an emancipation program. Two are from Morocco and one is from Guinea Conakry
Interview M.A.	Anthropologist-activist who has conducted a two-year ethnographic study with African youths transitioning to legal adulthood and has co-promoted an autonomous support network.
Interview R.C.	Social educator for young migrants in centers and activist involved with the Gender Equality Men’s Network of Sevilla and the autonomous support network for young migrants

### 2.2 Intervention phase

As opposed to any biological or sociological essentialism which links masculinity and violence, the objective of the X-MEN project was to try to bring about *mutations* in the awareness of underage boys who have been exposed to situations and contexts of violence from an early age, and who are therefore more likely to reproduce this behavior. Although gender-based violence is one of the central focuses of preventive action, in the case of minors migrating alone from Africa, we identified multiple levels of exposure to violence throughout the migration process that required attention during the workshops.

After the research phase, two cycles of 20-h workshops were organized in two different centers managed by Fundación SAMU.^7^ The first group consisted of 11 boys aged between 14 and 17 (seven of Moroccan origin, two from Guinea Conakry, one from Mali and one from Ivory Coast). The second group consisted of seven boys of Moroccan origin aged 15–17. The intervention was divided into two sequential phases: a first phase of reflection and a second phase of artistic expression. The methodology was based on the principles of dialogical pedagogy (Meirieu, 2007; Freire, 2023), focusing on conversation rather than explanation (Rancière, 2010), but also on participatory activities and settings inside and outside the center.

Intersectional feminism and intercultural dialogue were the guiding principles underlying the ethos of the intervention. Intersectionality invoked the development of critical awareness of the *locus of enunciation* (Ribeiro, 2020) and the *intersectional empathy* (Rodó-Zárate, 2021) between different discriminatory situations and groups of oppression, taking into account not only age, nationality, ethno-racial or religious oppressions, but also patriarchal oppressions based on a masculine and heterosexual position toward women (in their diversity and internal hierarchy) and the LGTBIQ+ community. Interculturality translated into “practicing enunciation from the same place where silence is imposed and with the voice of the silenced” (Adlbi Sibai, 2016, p. 270), which manifested itself, as we shall see, in intra-group racial conflicts, but also in the leading role of hip hop as a form of collective expression toward the host society.

We highlight the four main ideas that informed the training and that triggered the learnings we share:

- *Exploring the normative ideals of masculinity through sanction.* Using the group translation of the Moroccan expression *machirojola*^8^ (“that’s not manly”) as a starting point, we explored the motives behind the words used in the different mother tongues to say that a man *is not a man* or *is not man enough*, relating it to their actual experiences and, subsequently, to the ideals of femininity they expressed.
- *Stimulate awareness of their locus of enunciation.* Beginning by reflecting on the role that hip hop can play in terms of raising awareness, the type of awareness-raising and locus of enunciation (or structural position) were related through the analysis of video clips by artists such as Morad and Miss Raisa,^9^ which stimulated awareness of their location on “the hierarchical scale of the modern/colonial world-system” (Adlbi Sibai, 2016, p. 266).
- *Include the groups in activities with local people*. In an effort to address the isolation and ghettoisation of these minors (Empez, 2014; Martín-Sánchez et al., 2020), we attended an anti-racist event in a “safe space” in the city, where a documentary starring “menas” and “exmenas”^10^ was shown.
  *Establish contact with adult migrant community figures with anti-racist and anti-sexist sensibilities*. In the first group, this role was played by Marra Junior, a Senegalese anti-racist and pro-feminist activist^11^ and by Moroccan members of the Ex-Menas collective.^12^ In the Moroccan group, this role was played by the hip hop instructor, Fugi Alkayssar, a refugee artist of Algerian origin (Table 2).^13^

**Table 2:** Information produced during the workshop (intervention phase).

Group 1	11 boys aged 14–17 years old. Nationalities: Morocco: 7 Guinea Conakry: 2 Ivory Coast: 1 Mali: 1
Method	Discourses and materials produced by the boys during the sessions through dialogical techniques, such as mapping, brainstorming output, debates, conflict resolution assembly and the creation of a hip-hop song. Weekly dialogue with observers (main educator, mediator and Internship student) before the sessions. Some observation reports were shared through WhatsApp voice recording. Field diary (mainly voice recorded after the sessions). All this information, along with other data, was analyzed and contrasted in order to construct a narrative of the workshop, finally developed in the Evaluation System document.
Group 2	7 Moroccan boys aged 15–17 years old.
Method	Discourses and materials produced by the boys during the sessions through dialogical techniques, such as mapping, brainstorming output, debates, conflict resolution assembly and the creation of a hip-hop song. Interview with a female Spanish educator and a female Moroccan Mediator at the end of the workshop. All this information, along with other data, was analyzed and contrasted in order to construct a narrative of the workshop, finally developed in the Evaluation System document.

## 3 Results and discussion: learnings from the fieldwork

### 3.1 Law-border: protection, exclusion, and the politics of suspicion

The legal characteristics that define the condition of “MENA” (unaccompanied foreign minor) are the following: (a) being a minor; (b) the absence of a responsible adult; (c) immigrant status. According to European legislation, unaccompanied foreign minors are people under 18 years of age who are nationals of third countries and who arrive in the territory of the Member States of the European Union unaccompanied by an adult who is responsible for them.^14^ The legal framework for the protection of children, both at international, European and national level, provides a certain legal, and therefore crucial, security for minors arriving alone in Spain. However, this protection is undermined by provisions linked to migration policy, such as procedures “relating to the initial reception of children and their identification and registration by the police authorities; age determination; and, finally, the question of access to a residence permit” (Ceriani Cernadas, 2021, p. 18).

As UNICEF Comité Español (2019) points out, migration laws regulate the main aspects of the treatment of unaccompanied migrant minors in much greater depth than child rights protection laws. This results in certain limitations of the regulations on children and in some contradictions observed in the application procedures of the legal framework. It is first and foremost at the European Southern Border, in this case the Spanish border, where the first violations of rights upon arrival on the continent are evident, since this is where the *refoulement à chaud* takes place, i.e., the “return in the heat of the moment” to Morocco of all foreigners who have crossed the border, regardless of their age and without any legal guarantees (Premat and Moral, 2023). The European border regime has hardened since the 2015 migration crisis with the intensification of border controls and the securitization of migration policies, which have undermined migrants’ access to protection and basic rights (Hess and Kasparek, 2017). The authors (Hess and Kasparek, 2017) further argue that the logic of borders has intensified and expanded, with the border becoming the primary instrument for responding to migratory movements.

The system of guardianship for minors who migrate alone presents certain limitations as well, particularly with regard to obtaining documents which allow them to reside legally in Spain, such as the long duration of the procedures for issuing a valid identity card when minors are undocumented. On the other hand, serious incidents have been reported in relation to young people who have been under the guardianship of the autonomous communities and who are evicted from protection centers once they turn 18, even if they have not been documented or have not yet received a residence permit. In these cases, these “ex-minors” are forced to live on the street, homeless and undocumented (Accem, 2022).

Besides the legal definition and protection, the situation of these people is also the result of a social construction that is increasingly capable of obscuring the complexity that each minor embodies, with his or her personal, social and educational baggage. Jiménez and Vacchiano (2011) underline the fact that these minors constitute a unique category: they are both minors who are subjects of law and foreigners who are objects of the culture of suspicion. International and national “child protection” regulations stipulate that all legally unaccompanied minors should be placed under the protection of the state; however, the regulations governing foreigners establish them as persons to be monitored and repatriated. They are thus seen as two almost opposing phenomena: minors to be protected and unauthorized migrants to be expelled. The presence of unaccompanied immigrant minors in European territory highlights the contradictions of Spanish and EU migration policy and of asylum and refugee policy in relation to child protection systems, as well as the controversy over the externalization of European borders, which makes journeys to Europe increasingly dangerous, with fewer guarantees of basic human rights and with exposure to different types of violence.

The legal-political category of immigrant minors under the protection of the state was invented as a way to ensure equality of rights and, therefore, equal opportunities when compared with Spanish minors. However, at the same time, immigrant minors are subject to an inequality in terms of origin which makes this equality impossible. This category into which these young people are placed has not been accompanied by the establishment of other mechanisms which could help them in their incorporation into society. As a result, we have seen a kind of legal suspension which has resulted in deadlock, insecurity and marginalization, preventing these young people from building dignified lives for themselves (Calvo and Shaimi, 2020, p. 125). This sort of limbo is built around bureaucratic processes connected to the coming-of-age phase, like the age assessment procedure or the residence permit applications. Such waiting experiences can be particularly challenging for minors, as they often face uncertainty about their future and the possibility of deportation (Khosravi, 2021).

### 3.2 Age-border: children/men

Unaccompanied migrant minors share a dual liminal status in the receiving countries: that of adolescent and that of immigrant.^15^ Liminality is the threshold or margin of a social border that must be crossed by stripping oneself of all status in order to ascend to a higher one within a social structure, mediated through a rite of passage. Adolescent male migrants move from one social structure to another at the State level, but also within the structure at the World-System level (Wallerstein, 2012). And yet, *the immigrant* and the *adolescent* never seem to finish crossing the border, trapped in a permanent *rite of passage without passage*:

“It is no coincidence that migrants and adolescents are given an active or present participle as a denomination, precisely to underline the fact that they are condemned to remain constantly in transit, moving between states, without the right to repose” (Delgado, 1999, p. 112).

Unless or until they can prove that they are minors, they will first be dealt with according to the hostile approach of the law on foreigners, usually after having been subjected to a lengthy series of hostilities already.^16^ Lamine,^17^ a 19-year-old from Guinea who was previously under guardianship, compares situations of detention before and after crossing:

“In European countries they fingerprint you; in Africa they do not, but they put you in jail and then they take you out. Well, here they also put you in a prison, because if they put you in a room locked up alone, that’s a prison.”

Mohamed, an 18-year-old Moroccan who was previously under guardianship, points to his confinement in the Las Palmas center as the worst part of the journey, while his companion Aziz claims to have been attacked by police and social workers in Ceuta: “I have been beaten three times, once in the center for minors and twice in the port of Ceuta.”

Another age border in terms of the liminal masculinities of migrant minors involves being blocked from assuming the role of provider. According to Walker and Gunaratnam (2021), the imposed vulnerability of the dependent child subject can be perceived as emasculating for young men, as it requires a loss of autonomy, which contradicts their migration experiences and personal histories. When asked about the use of the expression *machi rojola* (“that’s not manly”), all the Moroccans agreed that “not working” can be a reason for receiving this insult. Both in Morocco and in Central and West African countries, a boy can begin to be called upon to assume adult roles from the age of 10 (Ghannami and Jiménez, 2018), with the consequent recognition of agency and capacity to assume the responsibilities of provider and family protector. Precocity is functional to the family and individual survival strategies that drive boys to emigrate, even when there is family opposition. This was the situation described by Lamine (19 years old), a Guinean who, at the age of 14, phoned his parents from Senegal to tell them about his plans to migrate, to the anger of his father and the support of his mother: “you are a man now, you have to fight, you have to earn a living*.”* This can also be seen in responses from the Moroccan focus group when asked about the role of men in Morocco:

- “For me, the word “man” means a person who is responsible for his life, not only for his own life, but also for the life of his family and those around them.” (Fatima, 18)
- “A man is not a strong thing with a beard. If there is no man in the family, the mother is the man of the house.” (Saloua, 17)
- “For example, boys who sell to help the family are men.” (Wafa, 17)
- “Some have two sides: one as a man who works for the family and another as a boy who goes to school and plays.” (Ali, 21)
- “If the father dies and there are older daughters, even if the son is 10 years old, we say: “you are the man of the house,” because there is always someone to protect the house and the family.” (Youssef, 17)

These same ideas are found in other studies on masculinity and gender relations in Morocco: “To be a man is to be responsible. And as I have to take care of everything, I feel that I am a man” (Woman, 45) (El Feki et al., 2017, p. 100).

The gender norms are clear for the men in the Moroccan focus group: men *go-out-to-fetch* and women *stay-in-waiting*. The Moroccan men express resentment of the pressure and sacrifice involved with having to support the family financially (as if there were no privilege in this), but rather than taking it up at the political or trade union level, it leads them to victimize and blame women, who they see as having a more comfortable role, and as not having to worry about the future. Social pressure to marry, the obligation to fulfill the role of provider and economic pressure are factors which contribute to men perceiving (or at least portraying) women as a *battleground* where their success or failure as a man is decided.

The women in the group reject this simplification and challenge this image of *passivity-waiting*, expressing aspirations for greater autonomy and independence from men by means of education and professional development (Dialmy, 2017). However, the men recognize that women “work all day at home,” although working in the home and childcare “does not fit the image of a man.” In other words, they blame them, but would not want to be in their place. In fact, the male mandate to *go-out-to-fetch* versus the female mandate to *stay-in-waiting* makes it less transgressive for a male minor to migrate alone than for an older woman, as it corresponds to a sharp gender division in relation to mobility in space. As Dialmy (Iglesias, 2016) argues, sexual borders are spatial borders from an early age.

All the males consulted have acquired experience in various forms of work, receiving vocational training, learning trades, contributing to the family’s agricultural, livestock or artisanal production (“I have no education or anything, just working in the fields… since I was three years old”—Mohamed, 18) or working for third party businesses, both before their departure and during their journey, especially the boys from Central and West Africa during their passage through the Maghreb countries. Furthermore, they all say that they want to send money back to their families (although among Central Africans there is a greater emphasis on individual goals), a discourse which, in contrast, is never heard from young migrant girls and women, who are looking to break free from an oppressive context, receive training and find a stable job, often without family or social support (Empez, 2014). In fact, in many young migrants’ lives, “work primacy” (Canizales, 2023) is a common feature: the centrality of work —often low-wage and exploitative employment—decreases the possibility of nonwork activities. “Work primacy oppresses social incorporation prospects by limiting working-class persons’—including unaccompanied, undocumented youth’s—ability to develop networks in school, family, and community spaces and by limiting their participation in non-work-related activities” (Canizales, 2023, p. 1390).

However, research carried out in other parts of the world (Cantalapiedra and De Jesús, 2018) shows that not all male migration involves a type of labor migration. According to a Spanish social educator interviewed in Tetouan, based on her experience with young Moroccans on both shores, there is a two-fold expectation among men that does not just involve fulfilling the role of breadwinner/provider and head of the family. Many of them want to migrate and experience life as young people in the European fashion, accumulate capital and experience as young bachelors and return to Morocco to live an adult life married to a Muslim woman with whom to start a family. According to her account, the reasons why male minors and young people emigrate go beyond the transnational provider role: there are also cultural reasons, which are related to aspirations for life experiences that are repressed in the culture of origin.

Ironically, male minors who migrate alone may be driven to do so because of poverty and having to fulfill the role of provider from a young age, caught in the tension between re-infantilization and economic imperatives. Yet it is this very agency and autonomy that they lose and risk in their quest to achieve economic adulthood.

### 3.3 Color-border: carrying racism with them

Pre-intervention research was limited to the majority group: Moroccan minors. We must acknowledge an epistemological racist bias as we had underrepresented the voices of black migrant people from West and Central Africa. These voices appeared in the first group to receive the training, to the point of requiring an on-the-fly redefinition of the objective of the intervention within the general framework of seeking to prevent the reproduction of violence by minors who have suffered it.

Until that point, we had been focusing on racism from the perspective of “maurophobia” (anti-Moroccan sentiment) and *white* negrophobia (Martín, 2011; Aixelà-Cabré and Rizo, 2023), in terms of institutional, police, symbolic and interpersonal violence (Del Sol, 2013; Gómez-Quintero et al., 2021). We proceeded on the basis of a strategy involving the intersectional politicization of empathy (Rodó-Zárate, 2021), where the distress caused by the experiences of structural discrimination were recognized and taken as a point of empathetic connection with regard to the patriarchal violence experienced specifically by women and perpetrated by men. Connections were made between racism, male chauvinism, Islamophobia, maurophobia and aporophobia. We reflected on how differences, inequalities and discrimination against a social group are created on the basis of phenotypical (skin color, genitalia) or cultural (religion, language) traits, and this was reflected in the hip hop lyrics they produced.^18^

However, right from the first session, we noticed a marked asymmetry in the space between Mauro-Arab and black people within the group and within the reception center itself. For example, the former monopolized common resources and occupied the armchairs as a group, while the latter were dispersed in chairs at the back. This conflict remained latent in the center. Ironically, in this center for minors, in contrast to the image of initial reception centers as places for disciplinary confinement (especially in Ceuta and Melilla), there was an affectionate and hospitable atmosphere, where warmth and affection were encouraged. However, this policy of promoting conviviality and coexistence conceals a racial conflict that has its own historicity overlapping with that of European colonialism^19^ and which manifests itself with each new group that arrives in the centers for minors and reception flats. Moreover, it appears that the reception institutions make no allowances or provisions aimed at managing this conflict.

The trigger was an activity in which the minors gave an account of their migratory journey using large maps which contained the participants’ home countries. The Moroccans’ accounts were brief, mentioning that it had taken them 3 months at most to cross. But when a boy from Mali said it had taken him 3 years, some of the Moroccans laughed in surprise (“Three years!”), showing their lack of knowledge as regards the situations of their fellow Central and West Africans. As the story progressed, the young Malian connected with the pain of what had happened on the Algerian border to the extent that he burst out with: “They killed my friend like a dog!” The atmosphere of the session changed completely, with the black boys in the room bowing their heads as the Moroccans called for the session to end. All the black participants had suffered racist violence in Arab-Muslim countries.

Moroccans make the same racist distinction that we white people make when we say *Africa* to refer to black-majority countries (Teruel, 2016).^20^ Within the group, there seems to be a tension between racializing and religious categories, with “Arab” overriding “black” in terms of the image of Muslim men. The possible commonality (i.e., the Muslim community) is undermined by the racist construction of the *black person* as *otherness* and *Africa* as primarily *black* (Mbembe, 2016). Black boys described their accretions of racialisation as they journeyed to North African countries (Walker and Gunaratnam, 2021) as stated by the words of Hassan (Guinean, 17) in front of the group:

“I did not know I was black until I arrived in Algeria, until then I was just a normal boy […] What I cannot understand is how people who are also Muslims can treat us like that; that’s not what Islam says.”

It was also made explicit, with great indignation, that this violence is inflicted on them because they are black and vulnerable when a Moroccan boy tried to explain the reason for the violence:

Traoré (Ivorian, 17): “In my country there are whole neighborhoods of Arab families living there and they do not suffer from racism.”

Ali (Moroccan, 17): “That’s because they go to work, they buy a house and live there; you are just passing through.”^21^

During the sessions, as we noted in the research phase, it became evident that the Moroccans were reluctant to point out and condemn experiences of violence and inequality, unlike the black boys, who were eager to address the racial conflict within the group. “I experience more racism in the center than in the street,” said Traoré, singling out the Moroccan boys in the group. Abdel, a 14-year-old Moroccan, described his own predicament, but in the form of an invitation to self-sacrifice: “At school they said to me, *you shitty Moor, go back to your country*, and what can I do? Well, keep my head down and keep going.”

Ultimately, the reception system for male minors who migrate alone, centered on social assistance and the “final sprint” of employability, overlooks and undermines socio-educational and therapeutic needs that go beyond the search for employment and that can be sensed in the groups. There is a need to address the “migratory trauma” (Kilomba, 2023) of one of the weakest links in the racist chain: minors who migrate alone. This trauma is the product of a historical, structural and systemic violence that Europe benefits from with its biopolitical coldness. In each mixed group that is received, the North/South racial hierarchy—on both an African and global scale—crystallizes on the back of the liminal masculinities of migrant minors, with black minors occupying the lowest rung of the ladder.

The conflict situation experienced in this group was partly resolved by following one of the recommendations identified during the research, namely, that it is a good idea to have adult figures from their countries of origin and/or who have had first-hand experience of emigrating to Europe as Africans, as the drivers of the interventions and/or as collaborators. Each of them provided a legitimate and critical reference point for the boys. The clarification and mediation of the conflict, the commitment of the social workers, the proposal to hold monthly assemblies in order to discuss and resolve issues relating to cohabitation and the collective creation of an anti-racist and anti-sexist message through hip hop, all contributed to better cohabitation and a certain restitution of dignity.

### 3.4 Masculinity-border: between reception and the reproduction of violence

As we have pointed out, the main objective of the workshops was to reflect on masculinity and to get the young and adolescent participants to understand that they have the option of becoming men who distance themselves from hegemonic and violent practices, but who are also able to speak about their fears and conflicts.

The naturalization of the idea that men’s role is that of provider can also implicitly perpetuate other traditional ideas related to masculine traits and roles such as women’s subalternity, neglect of self-care and care tasks, emotional hardening, risk-taking behavior, instrumental use of violence, etc. (Homes Igualitaris - AHIGE Cataluyna, 2022). The relationship between masculinity and violence has been studied by several authors such as Mead (1981), Kaufman (1989), and Gilmore (1994). However, the relationship between masculinity and violence is not only a factor behind the violence suffered by women. Violence also occurs, and to a great extent, between men. Kaufman’s concept of the “triad of violence” (1989) includes three types of male violence: violence toward women and children, violence toward other men, and violence against themselves. Consequently, when we talk about working with minors to prevent violence, we cannot only focus on violence toward women or girls, but also on violence among the boys themselves, many of whom—as we have already pointed out—are victims of violence both during their journey and when they arrive in Spain.

Throughout the research-intervention process, different forms of expression of violence have been detected in the lives of the majority of minors who migrate alone. On the one hand, there is the violence they are likely to be subjected to or receive and, on the other, the violence they are likely to reproduce. Regarding the latter, we repeat that minor males who migrate alone are not a homogeneous group and, although they themselves do not read or explain their differences in political terms, one of their central points of difference lies in their political sensibility. This is why pro-egalitarian views were expressed in response to those who advocate racism or male chauvinism.

The *violence received* that was detected refers to three interconnected levels of violence: symbolic, structural, and direct (Galtung, 1990). Meanwhile, in terms of *violence reproduced*, we find manifestations of racist, misogynist and homophobic symbolic violence, whether explicit or implicit. By symbolic violence, we mean: “any aspect of a culture that can be used to legitimize violence in its direct or structural form” (Galtung, 1990, p. 291; Bourdieu, 2000). We will not dwell on the forms of violence already mentioned in the previous sections.

### 3.5 Violence received

Both the focus group and the social workers interviewed agree that the principal form of violence—due to its normalization and daily occurrence—is the powerful psychological pressure in Moroccan culture for children to take on adult roles early on. The absence of a period in their lives where young people have their own visible time-spaces and where they can relate to their peers with autonomy, contrasts with the omnipresence of early pressure to create a family. Moreover, this is in a context with no job prospects, even for qualified young people (Recio and Gómez, 2011), and with a growing intergenerational gap between young and old (Desrues and Moreno, 2006).

Related to the above, the pressure placed on male migrants to achieve the ideal of success is noteworthy: all the accounts indicate that returning empty-handed is punished in the localities of origin. Returning without work or spending power is a source of shame and social guilt. Failure is seen as individual guilt. According to the Guinean Lamine (19), they are accused of being lazy and of having lived on benefits in idealized countries where everyone has work and money, idealizations often fed by the social network accounts of those who have already crossed the border or by other migrants who, on returning (to live or visit), prove or appear to be economically successful. The unsuccessful returnee is devalued as a man, while women are devalued when they emigrate.

In addition, both social workers highlight the issue of sexual violence against minors. The Moroccan social worker says that she deals with the issue in her work with minors without family support who are between 8 and 15 years old through active listening groups, where on some occasions a minor has expressed his “discomfort with an adult man.” This is a difficult issue to address due to the strong religious taboo (*haram*) against speaking publicly about sexuality and the inadequate development of child protection policies (Amnistía Internacional, 2023), which ultimately results in impunity for the aggressor and a feeling of guilt among the victims.

The Spanish interviewee said that young and adolescent males living on the streets are vulnerable to prostitution, where there is less demand from European women, who are seen as an opportunity to cross the border, and, above all, a demand from European men looking for minors, who are seen in the same way or as an opportunity to get money through blackmail, as homosexuality is punishable under Moroccan law. What is certain is that Morocco has become in recent decades a major destination for so-called “sex tourism,” with male prostitution a significant attraction, if not the predominant one.^22^

Furthermore, the Tetuan focus group, when asked about why men assault women in the context of a couple, develops a theory involving the reproduction and normalization of gender violence. They highlight the fact that these men have been on the receiving end of violence since childhood (which relates to the focus of the X-MEN project) and the possibility of early unconscious identification with adult men who engage in gender and sexual aggression. They also see gender violence as a product of a hierarchy of authority among men in public life, which would lead men who are subordinate to other men to demonstrate their power over women and children in the private sphere, in a kind of *patriarchal compensation.* They emphasize that it is not so much physical violence as everyday psychological violence, which is more subtle and normalized, including practices such as making someone—a woman or child—be *at the service of the man.* In the same vein, Marta Farré, after 2 years of ethnographic accompaniment with young Moroccans, said that they are young men who need to experience and learn how to establish non-hierarchical relationships. Once again, the group does not consider explanations based on biology or other types of essentialism which link masculinity and violence.

Finally, in addition to the racist violence already mentioned, we can identify from the interviews several forms of violence to which minors who migrate alone are specifically exposed. One of the Moroccans interviewed who had previously been under guardianship tells us that he spent 5 months living on the streets of Ceuta (Spain), where he and his fellow travelers were subjected to all kinds of aggressions, especially robbery (“They used to rob Moroccans who live in Ceuta; they have a red passport” [Spaniards of Moroccan origin], Abdel, 18). They felt completely helpless in the face of these robberies and other types of aggression, given their condition as people without citizenship or status (Agamben, 2010). The latter makes them more vulnerable to aggression, meaning that life on the streets consists of an endless loop of ongoing aggression and defencelessness.

Again the Guinean Lamine (19) points out a series of specific forms of violence experienced as a black migrant minor: hunger, long walks,^23^ returns to Moroccan borders (“it depends on your luck: if you can escape you go in, but if you get caught the police send you back”), short-term detention-punishment in prisons to dissuade them from continuing, assaults by border police (“they have dogs and they bite you”), witnessing violent deaths (“I saw a companion die on the skiff, I think because of fear,” “a boy died of an overdose”) and detention upon arrival in Europe.

Lamine also looks at problems relating to drug addiction, mental health and aggression that some minors get involved in as they attempt to cross the border, and how they are compounded by the lack of support and restrictions imposed on them as “minors” by the reception institutions:

“There are kids who set out with a certain mentality and the journey changes them. […] For example: in Morocco you had to smoke in the forest because it was very cold… They drank alcohol, took pills, smoked… they did all that for a year, then when you come here they tell you “you cannot smoke”: that creates problems, tension and people explode […] Some people take drugs, etc. on the way. They cannot do without taking anything anymore. So when they get here they lock them up and that’s a source of problems… Drugs, fights, etc. You have to see the state they are in because some of them have gone crazy because of the journey. It’s not a one-off event, it’s the whole dynamic.”

In fact, the minors themselves can become conveyors of xenophobic discourse due to the fear of “contagion” from the most problematic children in the centers:

“There are some guys who treat the workers very badly, but some of them are very good. There have not been any fights between us in a year, but I had a fight with a guy who was joking around at the beginning, but he broke my headphones. I’m not here to fight with anyone, you know? I’m here to work, to get my papers. What did you come here for? If you have come to make trouble, then just stay in your country” (Abdel, 18).

In our interview with Farré, who specializes as an anthropologist in individual/collective trauma and the body, she tells us that drugs such as hashish or anxiolytics are consumed for reasons of escapism (“they explicitly tell you so”), and in some cases this contributes to mental health problems. Farré examines the psychic suffering in the liminal masculinities of the migrant minors who have managed to cross. Firstly, she refers to the clash between the idealized Europe and the real Europe that they encounter. This discrepancy between the ideal of full European employment and the real situation involving barriers to access engenders frustration. Moreover, they are under pressure from two sources: from there, due to the expectations of success on the part of their families and communities of origin; and from here, due to rejection, confinement, exclusion, etc. Their self-image deteriorates as they internalize the construction of themselves as “poor,” “excluded,” “under care,” etc. All this is fed by a low emotional expressiveness, as they have to hide their weakness in order to survive in the hierarchical spaces they inhabit, despite the fact that in individual interviews (after many sessions) some of them spoke about having engaged in self-harm, suicidal thoughts and suicide attempts.

Lamine explains the key to successfully crossing the border: “what you need is a good heart.” By this he is not referring to some kind of philanthropic outlook, but rather to the idea of the body being able to withstand the violence and the most terrifying moments to which the migratory journey exposes you in these conditions. This is why he attributes the death of one of his companions on the boat in which he crossed to fear. When asked whether making the journey and achieving it makes you a hero, he replies “it does not make you a hero, it makes you a man,” underpinning the masculine ideal of sacrifice in relation to achievement. The experience of crossing the border exposes minors, as other migrants, to both vulnerability and militarized masculinity: direct violence by border guards and the police but also subtler forms of control and humiliation in situations in which migrants perceive a lack of power, especially shame and frustration from their own supposed failure to fulfill patriarchal norms and aspirations (Allsopp, 2017).

At the same time, there is a need to make sense of the sacrifice made, the violence endured and the suffering experienced. “Spirituality is what you are left with when you can no longer trust humans,” explains Farré. Spirituality, channeled through religion, is the inner strength that allows them to sustain the sacrifice. The only possible strategy when you are not in control is to trust in the gods, in destiny, so that whether you succeed or fail is not up to you: it was written. Fate, destiny, is divine design; as a human, one can only develop the capacity to withstand pressure and fear.

### 3.6 Patriarchal violence reproduced

As we have already said, here we will focus on the violence that the minors or young people we have come into contact with during the X-MEN project have reproduced on the symbolic level, i.e., as conveyors of hetero-sexist discourses, stereotypes or beliefs.

First of all, we will highlight some of the young men’s resistance and reactions to the assertions of strength and aspirations for equality on the part of the women in the discussion group, which are perceived as a threat of displacement (Desrues and Moreno, 2006). In response to the assertion by one of the women in the group that “a woman can be the man of the house,” as a patriarchal reaction, rather than referring to biology, a tautology is invoked: “A man can never be replaced in the family because a man is a man; he is important” (Youssef, 17). Defending this self-referential position of importance requires microviolences involving the undermining of women in the group (“She does not know what she wants”), the denial of gender inequality (“Men and women abandon the home on equal terms”) and the demonization of women (“There are widows who emigrate to rich Arab countries to do work of dubious morality,”^24^ “There are women who leave their unemployed husbands for another man”).

During the workshops, misogynist and homophobic positions were also expressed by some participants. In terms of misogyny, and without ethno-racial distinction, we detected conservative positions regarding men’s role as leaders in the home and the restrictions on women’s freedom when they become wives. In the following table, we summarize the answers given in response to the statements *Ali will be a man if…* and *Fatima will be a woman if…* from the point of view of the Moroccan migrant minors in the second group (Table 3).

**Table 3:** Male-female ideals according to a group of migrant Moroccan minors.

Ali	Fatima
Is a good Muslim Is not a faggot Makes a living by any means necessary Takes care of his family	Keeps the house clean Attentive, helpful Can be trusted Is faithful (x2)
Does not hit women Is not rude Is not a snitch Is not a liar Is not malicious	Does not talk to other men Does not bring other men into my house Does not tell her friends about it Is hot Is good-looking (x2)
Is reliable Is pure-hearted Is kind Is polite Is responsible Is fun Helps Loves his mother	Is “toxic”
Is tough (x2)	

The ethnocentrism of the two ideals is implicit, although the condition of “good Muslim” is only explicit for masculinity. The first four traits describe the main strands of traditional Moroccan hegemonic masculinity: Muslim, heterosexual (procreative), provider and protector. The negative definitions refer to trustworthiness between men. In the study, betraying this pact of trust between men emerged as one of the main reasons for saying *machi rojola*. Then come the positive definitions of this masculine honorability, which coexists with the toughness required in order to assume the assigned roles, but also with the desensitization required in order to be resilient in the face of the hostility of the situations in which they live.

However, their portrayal of women is in itself an exercise in patriarchal symbolic violence which often, as in the case of native Spanish adolescents, is intended as a defiance of the expectations of official educational agents. In local society, this defiance of educational authority is redefined by the ultra-right as “transgression of political correctness.” In the case of migrant minors, resistance is justified as self-defense against the assimilationist interference of European culture. Thus, for example, we find that a real woman is synonymous with “toxic,” provocatively insinuating to the social worker an essentialist link between woman and toxicity. However, as in the negative definitions of men, the portrayal of women is dominated by fear of other men and the influences of other women (female friends) and by the expectation of domestic servitude. In short, this ideal would be a woman who is at his service domestically and in terms of “taking care” (and by the insistence on the physical, we understand this as meaning sexually as well), and *stays-in-waiting,* isolated from all influences outside the home and marriage.

However, from the point of view of the Spanish social worker in northern Morocco, Moroccan youths make a clear distinction between “European women” and “Muslim women,” measured by two different standards of femininity. Practices seen as normal for European women (such as smoking in public spaces) are stigmatized in the case of Moroccan women (the “whore” stigma). They want to interact with European women, who represent freedom (including sexual freedom), but they do not want them to serve as a reference point for Muslim women. European women are seen as free women who do not make firm commitments, something which is required of Muslim women within the normative family model. However, although many migrant minors leave their countries with the idea that they will return as a success and form a Muslim family, according to the anthropologist interviewed, as time goes by, they begin to distance themselves from the Muslim woman as a model of attractiveness. Moreover, as mentioned above, it seems that this model is also being challenged and reshaped by Moroccan women in urban areas, who are in turn beginning to distance themselves from the traditional patriarchal model of Moroccan masculinity (Desrues and Moreno, 2006; Dialmy, 2009; Iglesias, 2016; El Feki et al., 2017).

Finally, there were different opinions expressed, as well as a great deal of silence, when it came to the issue of homosexuality. The workshop participant from Mali (Group 1) needed to check whether same-gender marriage is possible. He could understand that homosexual people had organized themselves and won rights, but he found it very strange that they would do it out of love. This has to do not only with homophobia and ignorance, but also with cultural differences around the institution of marriage, monogamy and romantic love. Regarding ignorance, Lamine from Guinea mentions the presence of couples of women or men in the streets as something that shocked him when he arrived in Spain:

- “I wasn’t used to a woman being with a woman, or a man with a man. But that’s OK, everyone has their own life and that’s that.”
- “Is it illegal in your country?”
- “I had not ever seen it; when I was a kid, I wasn’t aware of it, I did not know it was possible, but as my mind is already open a little bit because I’ve seen many things on the journey, I do not notice it, but the first day I saw it I felt I was really in another world.”

However, we also found more hostile attitudes on the part of the more religiously conservative boys who invoked *haram* (sin) to avoid even talking about the subject. This lack of information and homophobic denial makes it difficult to address the issue in specific interventions of short duration, but in both groups, the social workers told us about reactions of affection from one boy to another or of sadness after the session where it was discussed. It should not be forgotten that this may be one of the reasons for migration for a good number of these minors and that homophobic violence may be occurring as a matter of course, as we saw with racism, in the reception centers.

## 4 Conclusion

The main contribution of the article is the presentation of an intersectional methodology to address social intervention in violence prevention with unaccompanied migrant minors arriving in Spain. This methodology focuses on the consideration of liminal spaces as border and transition places that need to be named and taken into account for transformative work with these young people.

The liminal spaces are wide-ranging and diverse. For those of us whose work involves something between research and intervention, it is important that we consider the intersection of the different inequalities that affect them and the border situations in which many of them find themselves, whether legal, gender, age or racist borders, just to mention the ones we have dealt with in this article. Interventions which seek to address equality and the prevention of violence, and which have a transformative perspective in order to create egalitarian discourses and practices, must incorporate an intersectional gender approach which allows us to see beyond the effects of the, in general, stereotyped and stigmatized homogenization of these minors by the destination society. The one-dimensional view of these young people has the effect of creating a contradiction which is difficult to resolve: we construct them, from various points of view, as male providers—one of the strongest mandates of hegemonic masculinity—but, at the same time, they have to fulfill this role without being male chauvinists or violent. We used the concept of liminal masculinities in order to frame the way in which subaltern masculinity is incorporated by young migrants. Instead of providing a closed toolbox for how to be a man in Spanish society, we propose an intervention approach capable of integrating the youngsters intersectional locations as areas open to transformation and potential dynamism.

## Data availability statement

The raw data supporting the conclusions of this article will be made available by the authors, without undue reservation.

## Ethics statement

The studies and interventions involving minors were approved by the Commission for the protection of children and adolescents, Cepaim Foundation. The studies were conducted in accordance with the local legislation and institutional requirements. Written informed consent for participation in this study was provided by the participants or participants’ legal guardians/next of kin.

## Author contributions

VL: Writing – original draft, Conceptualization, Investigation, Supervision, Methodology, Writing – review & editing. BA: Conceptualization, Supervision, Writing – original draft, Methodology. ÁR: Conceptualization, Investigation, Writing – original draft, Methodology.

## Footnotes

^1^For more information on the project, see: https://xmen.ces.uc.pt/.

^2^The concept of total social fact is defined in the work of Marcel Mauss. Sayad revisits this proposal and applies it to migrations, movements that involve the political, economic, social, subjective and institutional dimensions of the spaces they traverse. The aim is to restore complexity to a phenomenon that cannot be understood without a multi-focal approach.

^3^The sociology of childhood inevitably suffers from adultcentrism, since the population group that falls within this construction (children, adolescents) does so due to adult assignment in a relationship of asymmetrical structural forces, which is not incompatible with the recognition of agency and resistance on the part of the *minoritised* group (Rodríguez, 2007). In this case, since we are dealing with migrant minors, epistemological ethnocentrism is also involved: we find how the categories inherited to designate age are often not in line with the socio-cultural realities of the countries of origin in Africa, regardless of biological and cognitive evolution. For this reason, although terms such as “migrant children,” “minors,” “children” or “adolescents” are used, we wish to do so while being aware of this two-fold adult-ethnocentric misalignment.

^4^Connell’s conceptualizations help us to analyze gender relations on the basis of three dimensions: relations of production, which have been altered with the massive incorporation of women into the labor market and the public space; relations of power, where violent behavior is associated with a supposedly masculine essence; and relations of affection, emotions and sexuality, which are impacted by the growing disconnection of sexuality from reproduction. These dimensions would be the components of any gender order or gender regime.

^5^ https://www.congreso.es/public_oficiales/L14/CONG/DS/CO/DSCD-14-CO-557.PDF

^6^Information on the program: https://cooperacion.us.es/sites/default/files/2022-03/Publicidad-IV-Coloquio_3%C2%AA-convocatoria.pdf.

^7^ https://www.samu.es/infancia-y-familia/

^8^The pedagogical use of the expression is inspired by the feminist podcast *MachiRojola,* produced by the Ellile collective in Casablanca. Boushaba (2021): *The podcast that seeks a new inclusive masculinity in Morocco*, inEldiario.es: https://www.eldiario.es/internacional/podcast-busca-nueva-masculinidad-inclusiva-marruecos_1_8071549.html

^9^Morad is a singer born in Spain of Moroccan parents, a sort of spokesman for the MDLR (*mecs de la rue*), even though he is not one of them. He deals with themes such as friendship, the neighborhood, street children, love for one’s mother and hatred of the police. Miss Raisa emigrated to Spain when she was 8 years old and is much less well known. She is a young Muslim girl who sings in protest against the gender-based Islamophobia she has suffered since childhood and in support of the empowerment of young Muslim women. She has been attacked on social media by both Islamophobes and conservative Muslims.

^10^Servicio de Producciones Audiovisuales de la Diputación de Cádiz (2022): “M.” https://www.youtube.com/watch?v=SwMAWnx5stw. Sara Ortega (20/01/2023): “M,” the documentary in which migrant youth speak.Público.https://www.publico.es/sociedad/m-documental-habla-juventud-migrante.html

^11^Elena García (02/01/2021) Marra Junior: “There are men who want to continue taking advantage of their privileges, but many of us are trying to break with all that.” El Salto. https://www.elsaltodiario.com/en-el-margen/marra-junior-hombres-aprovechandose-privilegios-pero-otros-muchos-estamos-intentando-romper

^12^Juan Torregrosa (19/03/2021) Ismail El Majdoubi, spokesperson for the Exmenas Madrid collective: “The process to integrate unaccompanied foreign minors in Spain is not working.” La Puerta de África: https://revista.puertadeafrica.com/index.php/2021/03/19/ismail-el-majdoubi-portavoz-del-colectivo-exmenas-madrid/

^13^Juan León García (10/10/2020) *Flee or die in prison: the Algerian rapper who sings of freedom*. El Salto: https://www.elsaltodiario.com/migracion/huir-o-morir-encarcelado-el-rapero-argelino-que-canta-a-la-libertad

^14^Council Resolution of 26 June 1997 on unaccompanied minors who are nationals of third countries (97/C 221/03).

^15^In fact, the invention of adolescence has an intimate relationship with colonial racism. The term “adolescence” was coined by Stanley Hall in his 1904 study. In his second volume (1907), the white supremacist, civilizing zeal is seen in statements such as the following: “As we are gradually putting the child-world into schools of the latest type, so the primitive men and women of the world are coaxed or constrained to take up the burden of the white man’s civilization, and those who cannot or will not are following to extinction the larger wild animals about them that resist domestication […] Most savages in most respects are children, or, because of sexual maturity, more properly, adolescents of adult size” (Hall, 1907, p. 659). Retrieved from: https://archive.org/details/adolescenceitsps02halluoft/adolescenceitsps02halluoft/page/n7/mode/2up?view=theater

^16^First-person accounts: Sakho (2013) and Traoré (2018).

^17^The names of the minors have been changed.

^18^Report from the intervention, including excerpts from the song: *¿Prejuicios a mí?—Proyecto X MEN para menores que migran solos* (Prejudice against me?—X MEN project for minors who migrate alone). Fundación Cepaim (2023a): https://www.youtube.com/watch?v=C2vIWUW3bJo. Videoclip with lyrics in Spanish and Dariya: *Machi Rojola (No es de hombres)—Proyecto X MEN para menores que migran solos* (*Machi Rojola* (“It’s not manly”)—X MEN Project for minors who migrate alone). Fundación Cepaim (2023b): https://www.youtube.com/watch?v=4sqzy_8X938.

^19^“The trope of backward, primitive, childlike but naturally endowed Africans did not begin with European racists. It began with racist Arab writers. This ambivalent bromide of dehumanization and resource-focused backhanded compliment was amplified to new decibels in the period of the European encounter with Africa, beginning in the fifteenth century.” Ochonu (2021): for accounts of racist violence, but also of support and aid in the Maghreb, see Traoré (2018).

^20^Teruel (2016), *To be born in Africa is to be unlucky. Moroccans refer to black people as “the Africans.”* Frontera D: https://www.fronterad.com/nacer-en-africa-es-tener-mala-suerte-los-marroquies-se-refieren-a-los-negros-como-los-africanos/. This same distinction is found in one of our references: Aixelà-Cabré and Rizo (2023) *Afro-Iberia (1850–1975). Enfoques teóricos y huellas africanas y magrebíes en la península ibérica*. Bellaterra. (Theoretical approaches and African and Maghrebi traces in the Iberian Peninsula).

^21^A Spanish journalist comments on the ambivalence of current racism in Morocco: “Because it is true that, until too recently, the term “Africans” was used to refer to people who were passing through, who belonged to a continent south of the Sahara; it is also true that today we celebrate belonging to Africanness in institutional artistic expressions.” Iglesias (2020a): *Things that are said half-heartedly.* El País. https://elpais.com/elpais/2020/06/07/africa_no_es_un_pais/1591553986_581515.html.

^22^Najib (2007): *Prostitution masculine: Les travailleurs du sexe.* La Gazette Du Maroc: https://www.maghress.com/fr/lagazette/13031. Iglesias (2020b): *Prostitution practices define a society.* https://elpais.com/elpais/2020/09/02/planeta_futuro/1599068195_962238.html. On Moroccan society’s social rejection of pederasty and prostitution of minors in recent years, Vallejo (2013): *Morocco breaks the pederasty taboo*. RTVE. https://www.rtve.es/noticias/20130807/marruecos-rompe-tabu-pederastia/732820.shtml

^23^Vargas (2020): *Morocco abandons migrants detained during Covid in the desert: “We walked for six days without food or water.”* Público. https://www.publico.es/sociedad/deportados-marruecos-argelia-marruecos-abandona-desierto-argelino-migrantes-detenidos-covid-andamos-6-dias-agua-comida.html.

^24^This statement seems to respond to the boomerang effect of the stereotyping of Moroccan women in Arab countries. Perejil (2022): Moroccan women protest against their stigmatization as prostitutes in other Arab countries. El País. https://elpais.com/internacional/2022-07-23/mujeres-marroquies-claman-contra-el-estigma-de-prostitutas-que-sufren-en-otros-paises-arabes.html
